# Impacts of loading thymoquinone to gold or silver nanoparticles on the efficacy of anti-tumor treatments in breast cancer with or without chemotherapeutic cisplatin

**DOI:** 10.1186/s12896-025-00958-6

**Published:** 2025-04-10

**Authors:** Soha Gomaa, Mohamed Nassef, Ahlam Abu-Shafey, Mona Elwan, Asmaa Adwey

**Affiliations:** Department of Zoology, Science Faculty, University of Tanta, Tanta, Egypt

**Keywords:** Gold, Silver, Nanoparticle-based therapies, Thymoquinone, Cisplatin, Anti-cancer

## Abstract

**Background:**

Nanotechnology has been greatly examined for tumor medication, as nanoparticles (NPs) serve a crucial role in drug delivery mechanisms for cancer therapy. In contrast to traditional cancer therapies, NPs-based drug delivery offers several benefits, including increased stability and biocompatibility, improved retention capabilities and permeability, as well as precise targeting.

**Aim:**

The objective of this study was to examine the tumor-targeting efficacy of Thymoquinone (TQ)–loaded gold NPs (AuNPs/TQ conjugate) or TQ–loaded silver NPs (AgNPs/TQ conjugate) in conjunction with the conventional chemotherapy agent cisplatin (CP) in Ehrlich ascites carcinoma (EAC)-bearing mice.

**Methods:**

The loading capacity of synthesized conjugates was characterized by UV-Vis spectra and transmission electron microscope (TEM). We used CD-1 mice with a peritoneal EAC tumor xenograft model that received oral administration of TQ, AuNPs, AgNPs, AuNPs/TQ conjugate, and AgNPs/TQ conjugate.

**Methods:**

EAC-bearing mice received daily oral administration of one of the following treatments for six consecutive days: TQ, AuNPs, AgNPs, AuNPs/TQ, AgNPs/TQ, AuNPs/TQ + CP, or AgNPs/TQ + CP conjugates. Eleven days after EAC inoculations, assessments were conducted to evaluate the total number of tumor cells, splenocytes, white blood cells (WBCs), C-reactive protein (CRP) levels, flow cytometric analysis of apoptosis in EAC cells, as well as the functionality of the kidney and liver.

**Results:**

EAC-bearing mice that received treatment with TQ, AuNPs, AgNPs, AuNPs/TQ, and AgNPs/TQ exhibited significantly enhanced anti-tumor activity and improved therapeutic efficacy. Our results further revealed that the combined synergistic approach of TQ’s anti-tumor properties, along with the efficient penetration abilities of AuNPs or AgNPs, led to a significant inhibition of the growth of tumor cells in EAC tumor-bearing mice. Moreover, the incorporation of CP into the AuNPs/TQ or AgNPs/TQ conjugates substantially augmented the anti-proliferative effects against EAC tumor cells, effectively overcoming resistance to chemotherapeutic agents. Furthermore, our data revealed that this combination resulted in an elevation of leukocyte counts, along with an increase in the absolute quantities of lymphocytes, neutrophils, and monocytes, thereby activating the immune system and reducing the inflammatory marker CRP. However, the restoration of splenocyte levels, which had been reduced due to EAC cell inoculation, required an extended period to return to baseline. Furthermore, the results indicated moderate alterations in the functionality of both the liver and kidney.

**Conclusion:**

To conclude, AuNPs, AgNPs, AuNPs/TQ, and AgNPs/TQ may hold great promise as potential nanoparticle-based therapies for cancer treatment. Additionally, provides numerous benefits compared to conventional cancer therapies, such as selectivity and minimal side effects. Additionally, AuNPs, AuNPs/TQ, AuNPs/TQ + CP, AgNPs, AgNPs/TQ, or AgNPs/TQ + CP can specifically target tumor tissues, suppress tumor growth, extend the lifespan of tumor-bearing mice, and minimize cytotoxic effects on normal tissues, relative to the administration of free CP alone. More research is needed to understand the mechanisms of these nanoparticle-based therapies in clinical and optimize their use as cancer therapies.

## Introduction

Cancer represents the most widespread illness, and the increasing rate of mortality associated with it poses a significant challenge to address in the future [28, 118, 130]. Chemotherapy is the most widely utilized treatment for cancer, with cisplatin (CP) being one of the most employed chemotherapy agents in the management of various solid tumors [49, 71, 116]. Currently, the resistance of cancer cells to cisplatin (CP) presents a considerable obstacle in the chemotherapy treatment of multiple types of cancer. The primary issues contributing to treatment failure include the multidrug resistance associated with conventional cancer chemotherapy, as well as its lack of selectivity and cytotoxic effects. Current treatment methods may encounter limitations regarding their effectiveness and potential side effects. Consequently, ongoing research efforts are dedicated to tackling these issues and discovering safer, more precise alternatives [139, 66]. Notably, there has been a growing interest in the application of nanotechnology in cancer treatment, leading to the advancement of various nanoparticles designed specifically for targeting tumors in cancer therapy. Therefore, the potential use of nanoparticles (NPs) presents a targeted approach for the detection, targeting, and management of cancer treatment, which may help mitigate undesirable side effects and decrease multidrug resistance [127]. Nanoparticle (NP)-based strategies have been extensively studied to mitigate the various side effects associated with chemotherapeutic agents while enhancing their anti-tumor efficacy through the specific targeting of cancer cells [38, 74]. This is attributed to the unique, disordered, and multipore-filled vascular structure of cancerous tissues, along with impaired lymphatic drainage, which results in increased permeability and retention effects. Nanotechnology-based drug delivery strategy can utilize these characteristics to enhance the accumulation of chemotherapy drugs within tumors while reducing their absorption by healthy cells [25]. The advantages of metal nanoparticle therapy compared to traditional treatments are remarkable, with gold and silver nanoparticles proving beneficial for both diagnostic and therapeutic purposes [99]. To effectively eliminate cancer cells, nanoparticles capture incoming photons and convert them into heat [32, 113].

In recent decades, the utilization of nanotechnology within the medical sector has grown considerably, particularly in the realms of diagnosis, treatment, and targeted therapy for tumors, enhancing safety and efficacy. Nano-carriers-based drug delivery systems have demonstrated considerable benefits in cancer therapy, such as enhanced pharmacokinetics, accurate tumor cell targeting, decreased side effects, and lower instances of drug resistance [100]. The design or selection of NPs for these systems typically depends on their properties and size, tailored to the specific pathophysiology of tumors. In cancer treatment, nano-carriers are engineered to specifically target tumor cells by leveraging the inherent properties of NPs and the strategic placement of targeting agents following their absorption. Subsequently, they release therapeutic agents to the tumor tissue to facilitate cell death. The drugs encapsulated within these nano-carriers may include conventional chemotherapy drugs and nucleic acids, thereby serving dual roles in both gene and cytotoxic therapies [30]. Moreover, for specific medications with low solubility, NPs offer an effective means for encapsulation and systemic administration [137]. NPs, owing to their dimensions and surface characteristics, improve the permeability and retention of drugs, which in turn extends the half-life of pharmaceuticals and facilitates their accumulation within tumors [22]. Furthermore, the targeting mechanisms utilized by these systems are essential in safeguarding healthy cells from the cytotoxic impacts of medications, thus reducing the negative side effects linked to cancer therapies.

Gold nanoparticles (AuNPs) have been widely researched and developed as promising carriers for various chemotherapeutic agents, including CP, due to excellent biocompatibility, significant tissue permeability, straightforward preparation process, minimal toxicity, and anti-angiogenic properties, along with their ability to interact with a variety of target biochemical molecules [29, 64, 83, 90]. AuNPs possess surfaces that can be readily altered with a variety of high-affinity functional groups, which aids in the transport of bioactive compounds, including cancer therapeutic agents. This modification enhances the delivery of these therapies to intracellular environments, thereby improving their effectiveness against cancer cells [87]. AuNPs have recently been employed in cancer immunotherapy as carriers for cancer antigens and immune adjuvants [75]. Following in vivo administration, these nanoparticles are naturally absorbed by immune cells, which subsequently improves the effectiveness of tumor antigens [79, 94] and immune adjuvants [80]. Additionally, silver nanoparticles (AgNPs) are recognized as significant nanomaterials among various metal nanoparticles. They have been extensively utilized in numerous biomedical applications, including anti-inflammatory, anti-angiogenic, and anticancer therapies. In the scope of of administering anticancer agents via AgNPs, numerous studies have demonstrated cytotoxic effects on human cancer cell lines, leading to loss of membrane integrity, oxidative stress, and apoptosis, which ultimately results in cellular damage [17, 65, 104]. The therapeutic effects of AgNPs have been linked to a significant enhancement in antioxidant activity, cytotoxicity, and the potential to combat acute myeloid leukemia [136].

The adverse effects and lack of selectivity associated with many chemotherapeutic agents often lead to the consideration of natural drugs, which are preferred for their better tolerability and lower toxicity risks [98]. Extensive investigations have been undertaken to explore the possible benefits of herbal remedies, including thymoquinone (TQ), in improving the effectiveness of cancer treatments and safeguarding non-tumor tissues from damage caused by chemotherapy [19]. TQ, the primary active ingredient present in the volatile oil extracted from Nigella sativa seeds [107], has shown the capability to suppress various characteristics associated with cancer, including proliferation of tumor cells, inflammation, the apoptosis of cancerous cells, tumor angiogenesis, as well as invasion and metastasis [21, 48]. Furthermore, it enhances the anti-tumor effects of various drugs while mitigating their toxic side effects [24]. A major obstacle to the anti-tumor effectiveness of TQ is its restricted bioavailability [85]. Consequently, nano-carriers have been developed to improve the drug’s bioavailability, effectiveness, and stability, and to reduce toxicity associated with high doses [10, 43], thereby enhancing its absorption by cancer cells [39, 102].

The formulation’s nanoscale size enables its penetration into cancer cells [96]. The low solubility of TQ in water can be addressed by encapsulating it within biodegradable NPs, which would not only enhance its bioavailability but also improve its thermal and light stability, thereby increasing its therapeutic efficacy and promoting its accumulation in the tumor [92]. This approach also promotes antioxidant properties and enhances pharmacological efficacy [76, 97]. Consequently, the purpose of this research was to examine the tumor-targeting efficacy of Thymoquinone (TQ)–loaded gold NPs (AuNPs/TQ conjugate) or TQ–loaded silver NPs (AgNPs/TQ conjugate) in conjunction with the conventional chemotherapy agent cisplatin (CP) in tumor-bearing mice model.

## Materials and methods

### Reagents

Thymoquinone (TQ; 200 mg/ml)) and Cisplatin, (CP; 1 mg/ml) were purchased from Sigma-Aldrich (Missouri, USA). The Ehrlich ascites carcinoma (EAC) tumor cell line was bought from the National Cancer Institute (Cairo, Egypt). RPMI-1640 media was bought from Invitrogen (USA), while 10× RBC lysis buffer was supplied by Invitrogen (California, USA). Tetrazolium MTT was got from ThermoFisher Scientific (Massachusetts, USA). Monoclonal antibodies for Annexin V and PI were obtained from Pharmingen (California, USA).

### AuNPs and AgNPs conjugate synthesis

Gold nanoparticles (AuNPs) and silver nanoparticles (AgNPs) utilized in this research were synthesized with the aid of oleic acid (OAc) and oleylamine (OAm) as capping agents, which served to inhibit particle aggregation, oxidation, and degradation, while also reducing surface hydrophobicity according to de la Presa et al. [36, 88]. To synthesize 100 mg of gold acetate (Au(ac)3) and 100 mg of silver acetate (Ag(ac)3), a mixture comprising 500 mg of 1,2-hexadecanediol and 30 ml of phenylether was prepared in a three-neck flask. This flask was equipped with a thermometer, a reflux condenser, and a nitrogen inlet to facilitate the synthesis process. Following the heating of the mixture to 80 °C, oleic acid (0.32 ml) and oleylamine (0.34 ml) were introduced, and the temperature was subsequently elevated to reflux at 260 °C for a duration of 30 min. Following this period, the heat source was removed, permitting the mixture to return to room temperature. The final product was precipitated by the addition of 40 ml of ethanol, and centrifugation was performed to eliminate the yellow-brown supernatant. The deep purple precipitate (in the case of gold) and deep yellow (in the case of silver) were then redispersed in 25 ml of hexane, incorporating 0.05 ml of oleylamine and 0.05 ml of oleic acid. The nanoparticles demonstrated exceptional long-term stability, remaining unchanged under typical environmental conditions for several days or even weeks.

The loading of TQ onto the synthesized AuNPs or AgNPs was conducted using olive oil as a medium. In this process, a mixture of AuNPs or AgNPs (3.6 ml), TQ (0.16 g), and olive oil (32 ml) was thoroughly combined through ultra-sonication for 30 min. The synthesized nanoparticle conjugates were characterized using UV-visible spectroscopy, subsequently followed by Transmission Electron Microscopy (TEM) using a JOEL JEM-2100 microscope operating at an accelerating voltage of 200 kV.

### Characterization of synthesized conjugates of AuNPs and AgNPs

The initial characterization of the laboratory-synthesized NPS was performed according to Tripathy [131] by utilizing a UV-visible spectroscopy instrument (Perkin Elmer, Lambda 25), in which the analysis of the peak absorption band was conducted using a scanning resolution of 1 nm. For this analysis, a 1 mL aqueous solution of nanoparticles was utilized. All samples were measured after being diluted tenfold, using a quartz cell with a path length of 1 cm. The morphology of the sample surfaces, encompassing the shape and average particle size, was analyzed through Transmission Electron Microscopy (TEM) with a JEOL-JEM-1200 (USA). The specimen was prepared by permitting the solvent to evaporate after applying a drop of colloidal solution onto a 400-mesh copper grid that was coated with an amorphous carbon sheet.

### Inhibition of tumor growth by AuNPs and AgNPs conjugates in vivo

The MTT assay was employed to evaluate the in vitro anti-cancer effects of conjugates of AuNPs and AgNPs on EAC cells, as reported by [53]. In brief, following collection, EAC cells were washed and suspended in a buffer of phosphate saline (PBS). The EAC cells were then cultured in RPMI-1640 medium (2 × 10^4^ cell /well) for 24 h. Subsequently, the conjugates of AuNPs or AgNPs, along with the reference drug CP at 10, 50, and 100 µg/ml, respectively were introduced into the wells, with three replicates for each condition. In each 96-well plate, three untreated control wells were incorporated, which contained either the media or 0.5% DMSO. These controls were established to provide a baseline for comparison against the treated samples.

The MTT assay was employed to assess the number of viable cells present after a 48-hour incubation period. The media in the wells were replaced with fresh media, and 10 µl of 12 mM MTT was added to each well. The plates were then incubated at 37 °C with 5% CO_2_ for 4 h. Following this, 50 µl of 0.5% DMSO was added to each well, and the wells were incubated at 37 °C for an additional 10 min. An aliquot of 85 µl was taken from each well, and the cells’ viability was assessed by measuring their optical density (570 nm) using a microplate reader (Bio-Rad, Japan).

The cell viability % was determined using the formula:

$${\rm{\% }}\,{\rm{viability = }}\left( {{\rm{AT - AB}}} \right){\rm{/ }}\left( {{\rm{AC - AB}}} \right){\rm{ \times 100}},$$

Where AT refers to the absorbance measured from the treated cells, AB signifies the absorbance of the blank sample containing only the media, and AC represents the absorbance of the control group consisting of untreated cells. The determination of IC_50_ was done by GraphPad Prism software (California, USA).

### Mice

Female CD1 mice, aged 6 to 8 weeks and weighing approximately 25 ± 2 g, were allocated into ten groups (*n* = 10). The experimental protocol was executed under the guidelines established by the Institutional Committee of Animal use and Care (ICAUC), Science Faculty, Tanta University, Egypt. Strict compliance with the ARRIVE guidelines was maintained throughout all procedures conducted during the research.

### Preparation of tumor model

EAC cells were sustained in an ascitic tumor format in naïve female mice through weekly intraperitoneal (i.p.) injections of 1 × 10^6^ cells per mouse, as outlined by [2, 53]. EAC cells were collected and quantified utilizing the Trypan Blue dye exclusion method. For the tumor model, 0.25 × 10^6^ EAC cells were challenged via i.p. inoculation into naïve female mice.

### In vivo experimental protocol

**Fig. 1:**
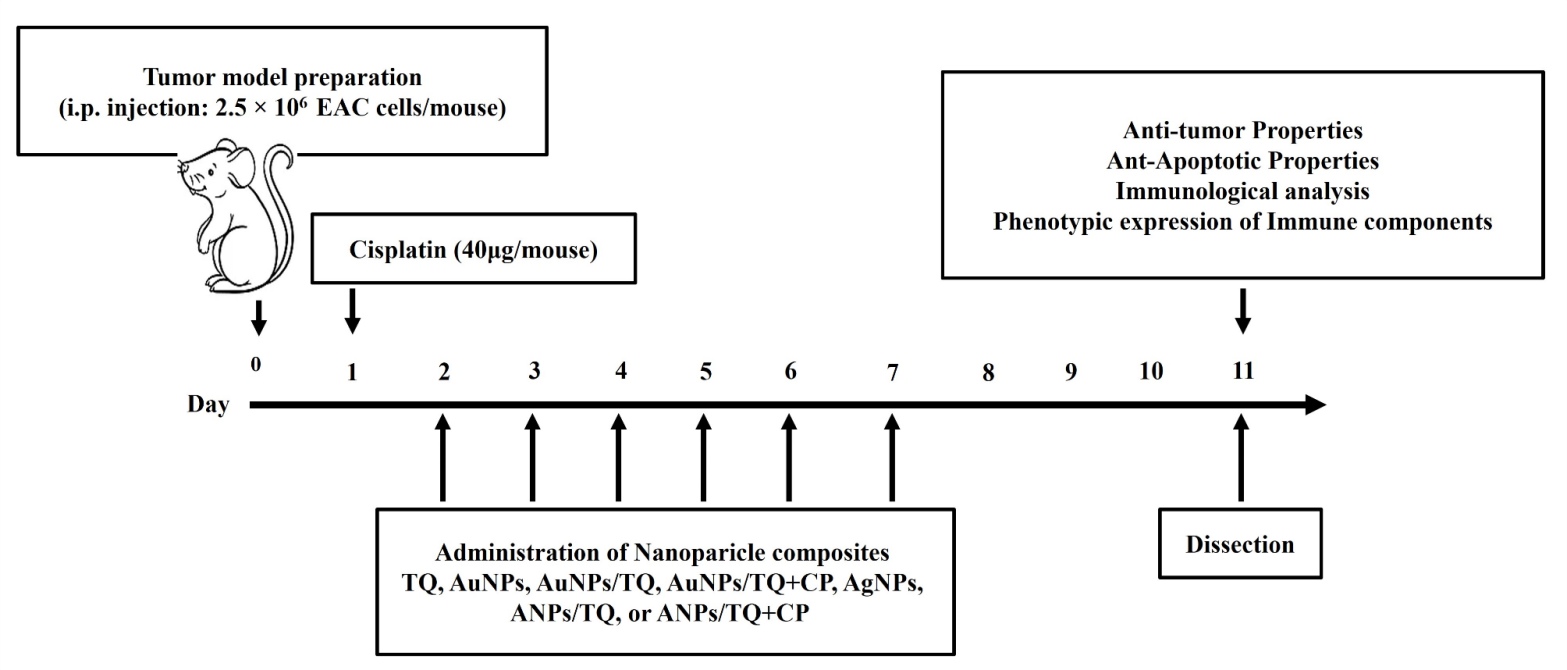
Schedule of the in vivo experimental framework and tumor challenge

The timeline of the in vivo protocol is depicted in Fig. 1. Ninety female mice were i.p. received 2.5 × 10^5^ EAC tumor cells/mouse and subsequently distributed into nine groups, each consisting of ten mice. On the second day post-tumor challenging, the 1st mice group received phosphate-buffered saline (PBS) (PBS group), the 2nd mice group received CP i.p at 1600 µg/kg mouse body weight (CP group), the 3rd mice group received TQ orally at 40 mg/kg mouse body weight (TQ group), the 4th mice group given AuNPs orally at 1332 µg/kg mouse body weight (AuNPs group), the 5th mice group administered AgNPs orally at 1332 µg/kg mouse body weight (AgNPs group), the 6th mice group orally received a combination of AuNPs at 1332 µg/kg mouse body weight and TQ at 1332 µg/kg mouse body weight (AuNPs/TQ group), the 7th mice group received orally a combination of AgNPs at 1332 µg/kg mouse body weight and TQ at 1332 µg/kg mouse body weight (AgNPs/TQ group), the 8th mice group i.p. received combination of AuNPs at 1332 µg/kg mouse body weight and TQ at 1332 µg/kg mouse body weight along with receiving CP orally at 1600 µg/kg mouse body weight (the AuNPs/TQ + CP group), and the 9th mice group i.p. received a combination of AgNPs at 1332 µg/kg mouse body weight and TQ at 1332 µg/mouse along with receiving CP orally at 1600 µg/kg mouse body weight (AgNPs/TQ + CP). All treatments occur once daily for six consecutive days. On the 11th day of tumor challenge, all mice were anesthetized with Isoflurane, and EAC cells, and sera spleens were gathered for future analysis.

### Harvesting and counting of EAC cells and splenocytes

As previously detailed by [52, 93], EAC cells and spleens were obtained from both treated and untreated tumor-bearing mice. The isolation of splenocytes involved dissociating the spleen using a 60 μm mesh sieve. Red blood cells were lysed using 10× RBC lysis buffer. The resulting cell suspensions were subjected to centrifugation, followed by washing and resuspension in PBS. Cell counts and viability assessments were performed by Trypan blue dye method.

### Evaluation of tumor cell apoptosis

EAC cells were obtained from both treated and untreated tumor-bearing mice, subsequently washed with ice-cold PBS, and suspended in a binding buffer of 1X annexin at 1 × 10^6^ cells/ml. For this cell suspension, 100 µL was combined with 5 µL of annexin V-fluorescein isothiocyanate and 1 µL of the PI working solution. Following incubation, a binding buffer of 1X annexin was introduced, and mixed gently, and subsequently, the cells were then subjected to analysis through a Partec flow cytometer (SysmexPartec Company, Germany). The phenotypic analysis of acquired samples was performed in FlowJo data analysis software (FlowJo, California, USA).

### Analysis of leucocytes and kidney and liver function

The serum levels of alanine aminotransferase enzyme (ALT), aspartate aminotransferase enzyme (AST), urea, and creatinine were measured calorimetrically with a fully automated biochemical analyzer (Vitalab Selectra E, Germany), utilizing standard ready-to-use kits (Human Gesellschaft für Biochemica and Diagnostica MBH, Germany). Blood samples were subsequently analyzed with an automated hematology analyzer (model MEK-6318 K, Japan) to assess various hematological parameters, including the total leucocyte count (10^3^/cmm) and the differential relative percentages of neutrophils, lymphocytes, and monocytes.

### Statistical analysis

All experiments were performed in triplicate. The data are stated as the mean ± SE from five independent measurements. Statistical analysis was performed as a one-way analysis of variance (ANOVA) as a part of an SPSS software package (v.16.0 for Windows, 2007; SPSS, Inc., Chicago, IL), and comparisons among groups were performed using post hoc Tukey’s HSD test, Dunnett’s test, and an independent sample t-test. A *p*-value < 0.05 was considered statistically significant.

## Results

### Characterization of AuNPs- and AgNPs- conjugates

**Fig. 2:**
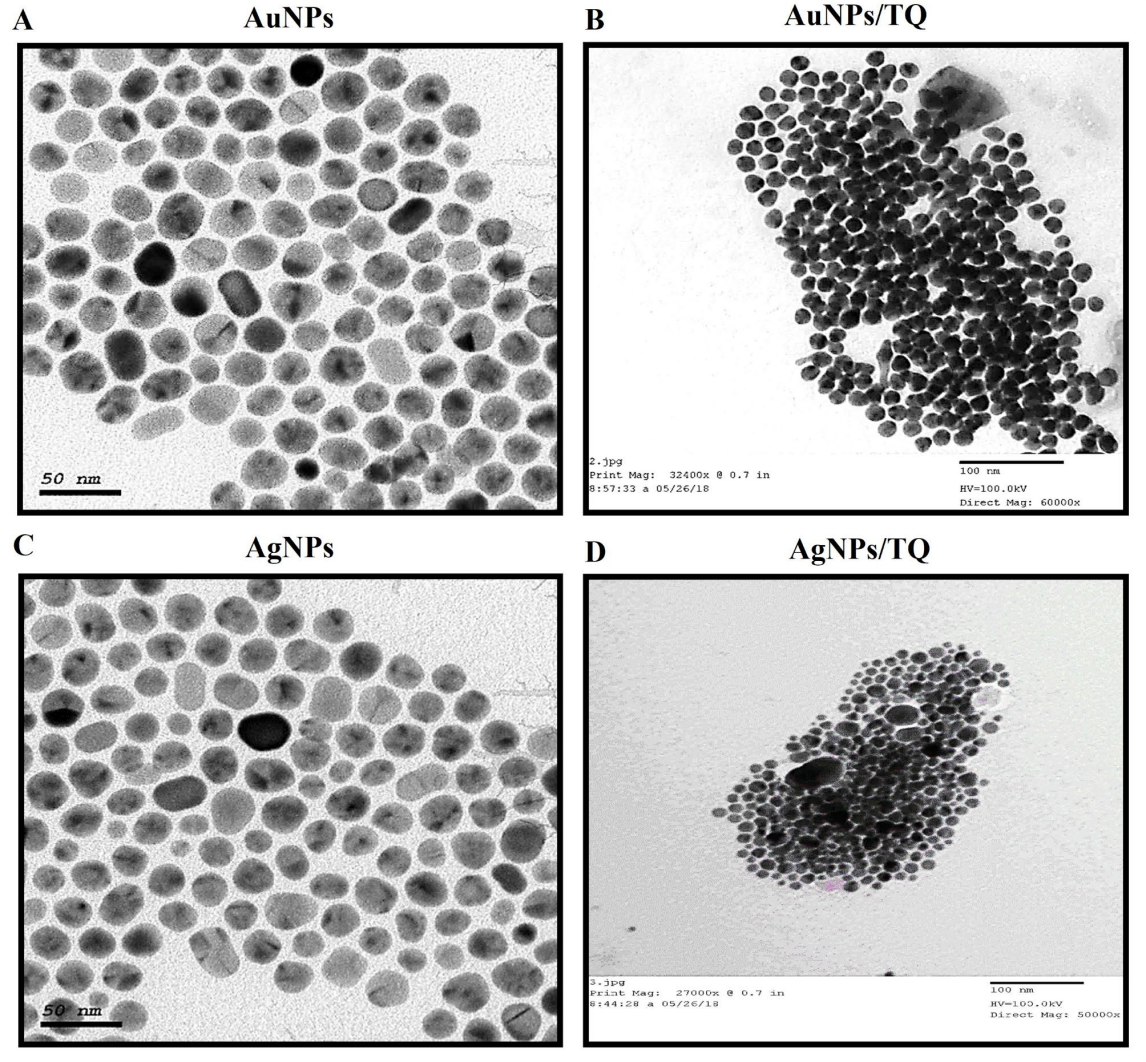
Characterization of AuNPs and AgNPs composites by TEM. (**A**) AuNPs, (**B**) AuNPs/TQ, (**C**) AgNPs, and (**D**) AgNPs/TQ

**Fig. 3:**
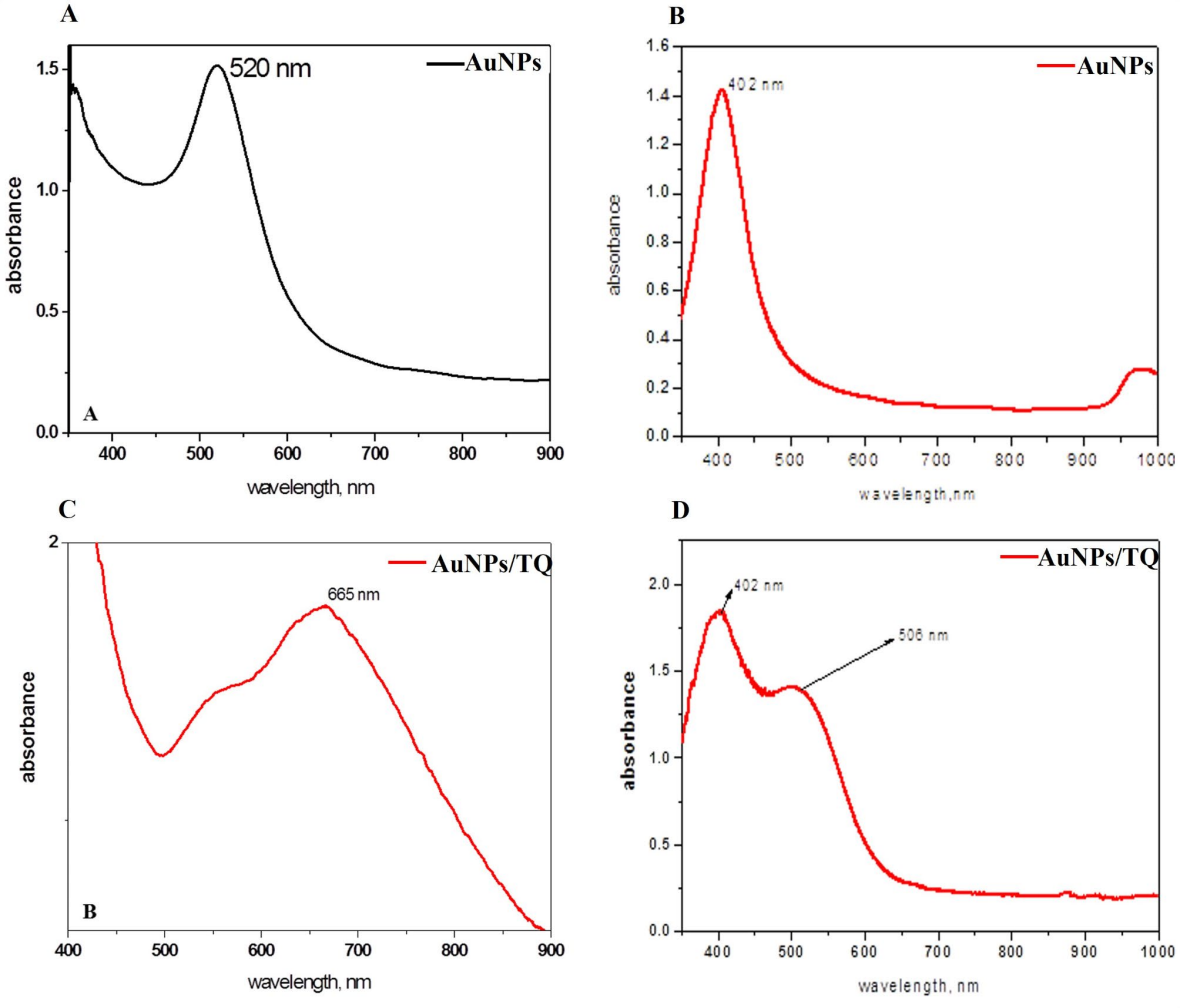
Characterization of AuNPs and AgNPs composites by UV- Vis characterization spectra. (**A**) AuNPs, (**B**) AuNPs/TQ, (**C**) AgNPs, and (**D**) AgNPs/TQ

AgNPs and AuNPs were successfully prepared and copped using OAc and OAm. The resulting AuNPs and AgNPs were loaded with 1 mg of TQ, and this loading was visually confirmed by a color change to pink-ruby red for gold and yellow for silver, indicating the reduction of gold or silver ions in the aqueous solution. The shape and size of the AuNPs and AgNPs composites were examined using TEM. The resulting AuNPs and AgNPs exhibited a spherical shape, as observed under TEM, and were well-dispersed without signs of agglomeration, with an average size of approximately 50 nm (Figs. 2-A, B). The morphology of the TQ-loaded AuNPs (AuNPs/TQ) and AgNPs (AgNPs/TQ) resembled that of spherical quantum dots (Figs. 2-C, D). UV-Vis spectroscopy provides a reliable method for assessing the AgNPs’ and AuNPs’ stability. This technique enables monitoring of their quality over time and the evaluation of the integrity of the colloidal solution during surface modifications. The absorption spectra of the produced AgNPs and AuNPs, consistently showed peaks at 520 nm and 402 nm, respectively, confirming the existence of AgNPs and AuNPs (Figs. 3-A, B). The incorporation of TQ into the synthesized AuNPs resulted in a noticeable shift in the peak at wavelengths of 665 nm and 506 nm, indicating the successful loading of TQ onto the AuNPs and AgNPs, respectively (Figs. 3-C, D).

### Inhibition of tumor growth by AuNPs and AgNPs conjugates in vivo

The in vitro impact of AuNPs- and AgNPs-conjugates on the proliferation of EAC cells and their growth inhibition was evaluated using the MTT viability assay. This assessment was conducted over 48 h following the introduction of the synthesized AuNPs- and AgNPs-conjugates to the EAC cells. IC_50_ values for the EAC cell line, measured 48 h after treatment, were determined to be 338.96 µg/ml, 30.88 µg/ml, 32.80 µg/ml, 63.29 µg/ml, and 62.02 µg/ml for TQ, AuNPs, AuNPs/TQ, AgNPs, and AgNPs/TQ, respectively (Table 1). The in vivo LD_50_ values were derived from the IC50 values, yielding estimates of 922 mg/kg (23 mg/mouse), 378 mg/kg (9.5 mg/mouse), 387 mg/kg (9.7 mg/mouse), 494 mg/kg (12.4 mg/mouse), and 490 mg/kg (12.3 mg/mouse) for TQ, AuNPs, AuNPs/TQ, AgNPs, and AgNPs/TQ, respectively (Table 1). A sublethal dosage was established for AgNPs and AuNPs, resulting in the calculation of i.p. doses set at 1 mg per mouse for TQ and 200 µg per mouse for AuNPs, AuNPs/TQ, AgNPs, and AgNPs/TQ (Table 1).

**Table 1:** In vitro Inhibition IC_50_ values (µg/ml) and in vivo LD_50_ (mg/kg) of AuNPs/TQ, AgNPs/TQ composites on EAC cell line

NPs composites	In vitro IC_50_ (µg/ml)	In vivo LD_50_ (mg/kg)	In vivo sub-lethal doses (mg/mouse)
TQ	338	922	1.00
AuNPs	30	378	0.2
AuNPs + TQ	32	387	0.2
AgNPs	63	494	0.2
AgNPs + TQ	62	490	0.2

LD_50_ values were calculated from In vitro IC_50_ according to the equation: log (LD_50_) = 0.372 × log (IC_50_) + 2.024 (Halle 1998, 2003)

### In vivo anti-cancer activity of AuNPs-, AgNPs-conjugates

**Fig. 4:**
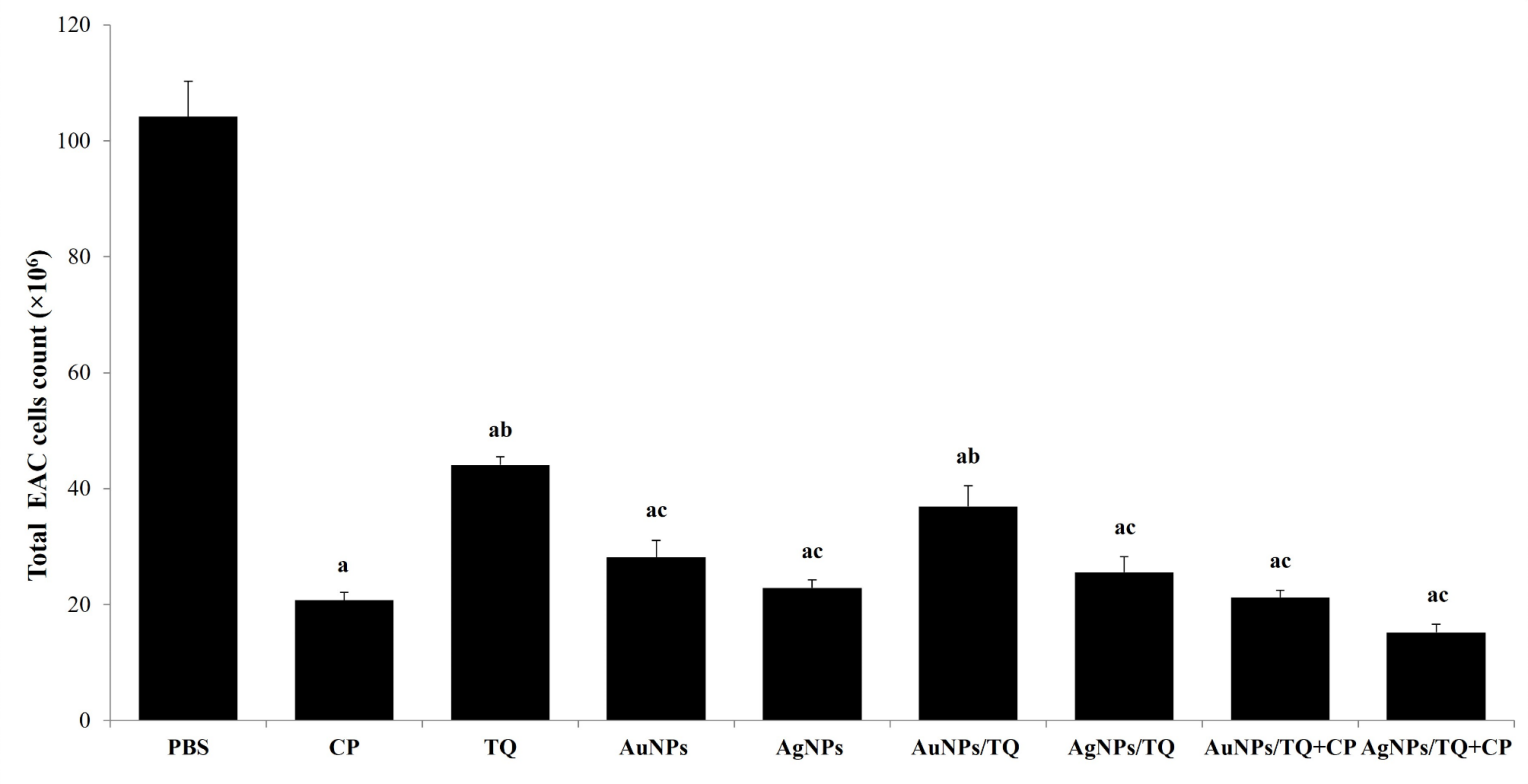
In vivo anti-tumor activity of AuNPs or AuNPs composites on EAC tumor-bearing mice. EAC tumor-bearing mice received PBS, CP, TQ, AuNPs, AgNPs AuNPs/TQ, ANPs/TQ, AuNPs/TQ + CP, or ANPs/TQ + CP daily for 6 successive days. The euthanasia of the mice occurred on the eleventh day subsequent to the inoculation of tumor cells. EAC cells were collected to assess their viability through the trypan blue method. Data are stated as mean ± SE (*n* = 5). A statistically significant difference between the groups was established at *P* < 0.05. ^a, b,c^ indicate statistically significant differences when compared to the EAC tumor-bearing mice receiving PBS (a), EAC tumor-bearing mice receiving CP (b), EAC tumor-bearing receiving TQ (c)

The data presented in Fig. 4 demonstrate that EAC tumor-bearing mice receiving CP, TQ, AuNPs, AuNPs/TQ, AuNPs/TQ + CP, AgNPs, AgNPs/TQ, or AgNPs/TQ + CP conjugates exhibited a significant drop in the number of EAC cells (20.8 × 106 ± 1.35, 44.1 × 106 ± 1.40, 28.2 × 106 ± 2.90, 36.9 × 106 ± 3.50, 21.2 × 106 ± 1.26, 22.86 × 106 ± 1.38, 25.56 × 106 ± 2.6, 15.2 × 106 ± 1.4, respectively) relative to naïve mice to EAC tumor-bearing mice group that received PBS (104 × 106 ± 6.10). Notably, EAC tumor-bearing mice receiving AuNPs/TQ + CP or AgNPs/TQ + CP conjugates achieved a reduction in the total number of EAC cells that was equal to or lower than that observed with CP treatment. Conversely, treating EAC tumor-bearing mice with TQ, AuNPs, AuNPs/TQ, AgNPs, or AgNPs/TQ resulted in a minor elevation in the count of EAC cells relative to naïve mice to those receiving CP. Furthermore, EAC tumor-bearing mice received AuNPs, AuNPs/TQ, AuNPs/TQ + CP, AgNPs, AgNPs/TQ, or AgNPs/TQ + CP conjugates significantly decreased the count of EAC cells relative to EAC tumor-bearing mice received TQ (Fig. 4).

### Splenocyte collecting and investigation

**Fig. 5:**
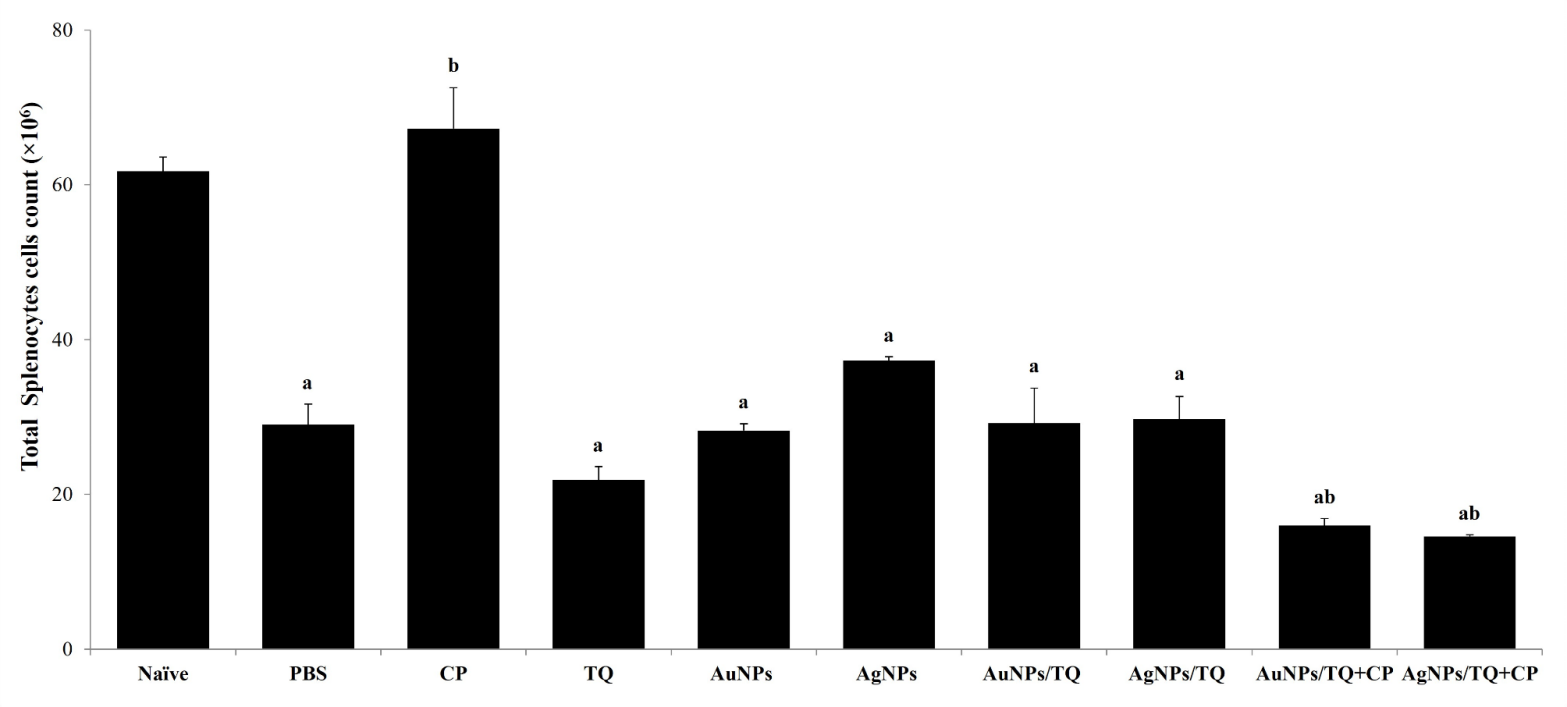
Potentials of AuNPs or AuNPs composites on the total count of splenocytes in EAC tumor-bearing mice. EAC-bearing mice were treated with PBS, CP, TQ, AuNPs, AgNPs AuNPs/TQ, ANPs/TQ, AuNPs/TQ + CP, or ANPs/TQ + CP daily for 6 successive days. The euthanasia of the mice occurred on the eleventh day subsequent to the inoculation of tumor cells. Splenocytes were collected to assess their viability through the trypan blue method. Data are stated as mean ± SE (*n* = 5). A statistically significant difference between the groups was established at *P* < 0.05. ^a, b,c^ indicate statistically significant differences when compared to the naïve mice receiving PBS (a), EAC tumor-bearing mice receiving BPS (b)

As illustrated in Fig. 5, treating EAC-bearing mice with PBS, CP, TQ, AuNPs, AuNPs/TQ, AuNPs/TQ + CP, AgNPs, AgNPs/TQ, or AgNPs/TQ + CP conjugates led to a noticeable drop in the total number of splenocytes relative to naïve mice (29.02 × 106 ± 2.64 × 106, 67.25 × 106 ± 5.31 × 106, 21.85 × 106 ± 1.73 × 106, 28.23 × 106 ± 0.90, 29.20 × 106 ± 4.55, 15.97 × 106 ± 0.92, 37.30 × 106 ± 0.51, 29.74 × 106 ± 2.93, 14.54 × 106 ± 0.25, respectively, versus 61.76 × 106 ± 1.82 × 106). Notably, treating EAC tumor-bearing mice with CP caused a remarkable elevation in the total splenocyte count (67.25 × 106 ± 5.31 × 106). In contrast, treatment with AgNPs or AgNPs/TQ resulted in a slight increase in splenocyte counts (37.30 × 106 ± 0.51, 29.74 × 106 ± 2.93), while treatments involving TQ, AuNPs, AuNPs/TQ, AuNPs/TQ + CP, or AgNPs/TQ + CP did not significantly alter or marginally decreased the total splenocyte count relative to EAC tumor-bearing mice treated with PBS (29.02 × 106 ± 2.64 × 106) (Fig. 5).

### Apoptosis assessment

**Fig. 6:**
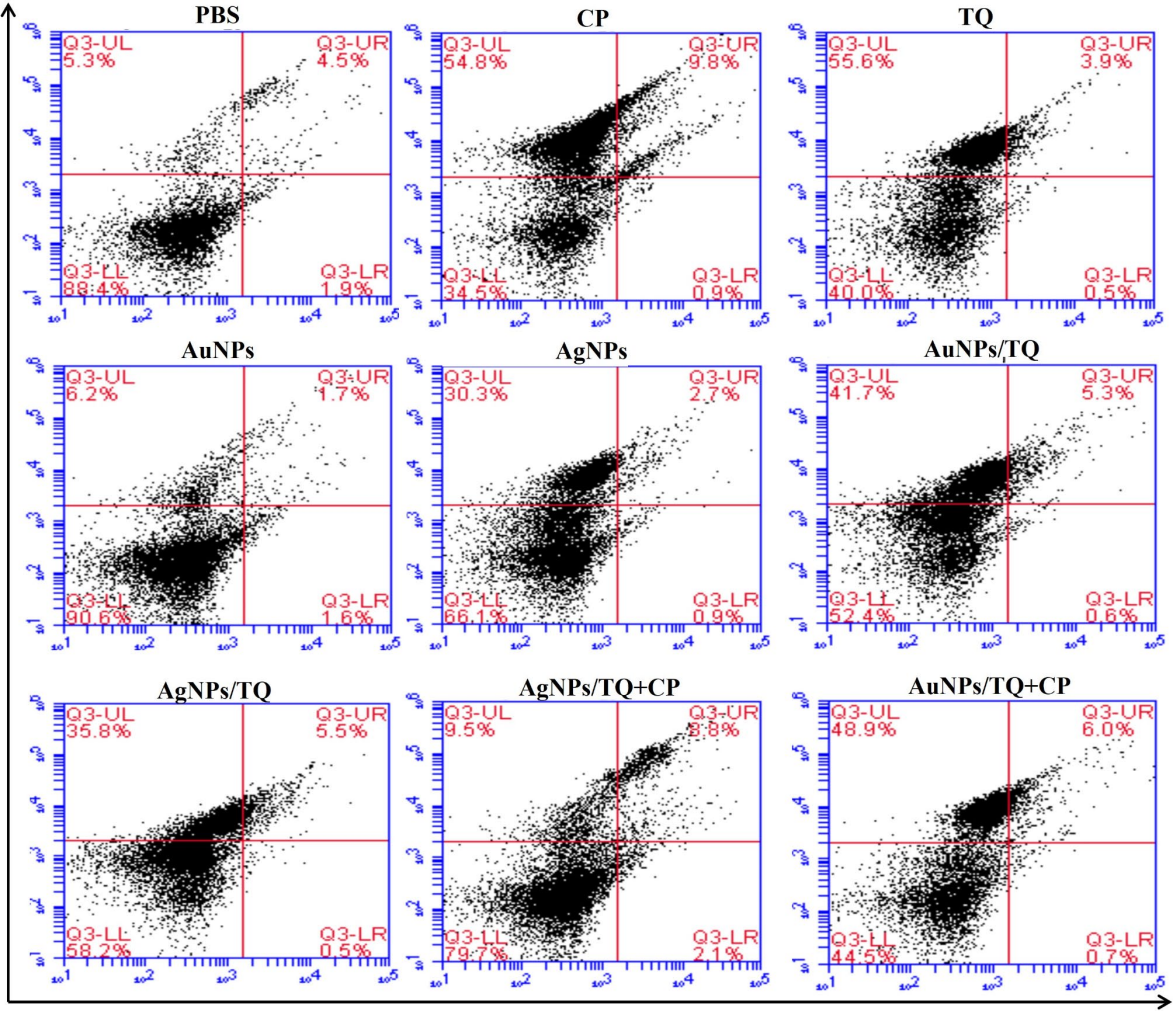
Phenotypic analysis of EAC cells in EAC tumor-bearing mice receiving AuNPs or AuNPs composites. EAC-bearing mice were treated with PBS, CP, TQ, AuNPs, AgNPs AuNPs/TQ, ANPs/TQ, AuNPs/TQ + CP, or ANPs/TQ + CP daily for 6 successive days. The euthanasia of the mice occurred on the eleventh day subsequent to the inoculation of tumor cells. Ascitic EAC cells were collected from peritoneal cavities. EAC cells were subjected to staining with Propidium Iodide (PI) and annexin V, followed by an assessment using flow cytometry to analyze the specified marker as illustrated in the representative histograms

**Fig. 7:**
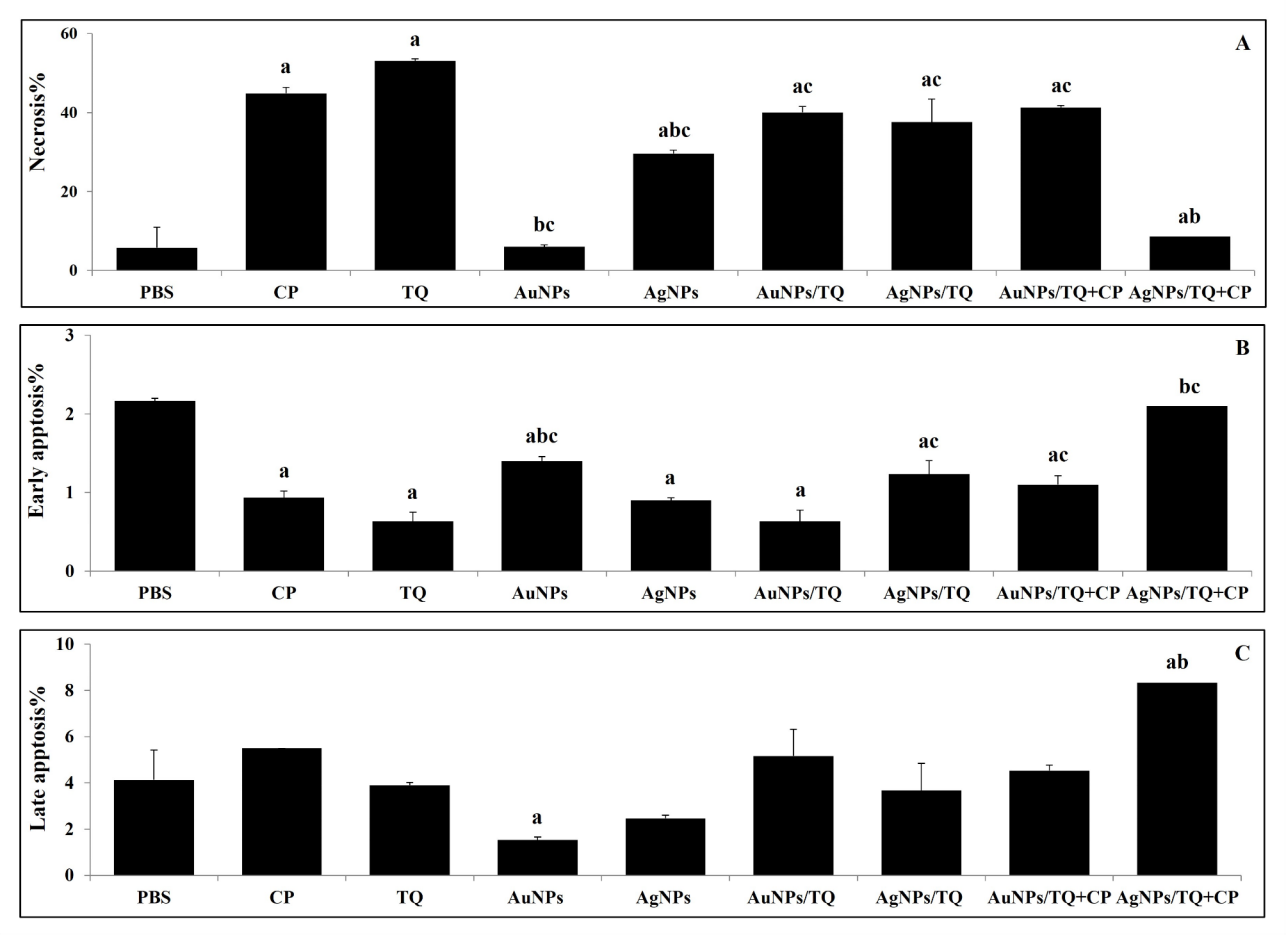
Necrotic potentials of AuNPs and AuNPs composites on EAC cells in EAC tumor-bearing mice. Necrosis (**a**), early apoptosis (**b**), and late necrosis (**c**). EAC tumor-bearing mice were treated with PBS, CP, TQ, AuNPs, AgNPs AuNPs/TQ, ANPs/TQ, AuNPs/TQ + CP, or ANPs/TQ + CP daily for 6 successive days. The euthanasia of the mice occurred on the eleventh day subsequent to the inoculation of tumor cells. EAC cells were collected to assess necrosis, early apoptosis, and late apoptosis. Data are stated as mean ± SE (*n* = 5). A statistically significant difference between the groups was established at *P* < 0.05. ^a, b,c^ indicate statistically significant differences when compared to the EAC tumor-bearing mice receiving PBS (a), EAC tumor-bearing mice receiving CP (b), EAC tumor-bearing receiving TQ (c)

The data presented in Fig. 6 indicate that treating EAC tumor-bearing mice with CP, AuNPs, AuNPs/TQ, AuNPs/TQ + CP, AgNPs, AgNPs/TQ, and AgNPs/TQ + CP, resulted in a reduction in the necrosis percentage (5.77%, 5.97%, 40.03%, 41.27%, 29.60%, 37.60%, and 8.57%, respectively). Particularly, the inoculation of TQ caused an increase in treatment efficacy, achieving a necrosis percentage of 53.10% relative to 44.87% in EAC tumor-bearing mice receiving PBS (Fig. 7A). Furthermore, treating EAC tumor-bearing mice with CP, AuNPs, AuNPs/TQ + CP, AgNPs, AgNPs/TQ, or AgNPs/TQ + CP caused an increase in early apoptosis rates (2.17%, 1.40%, 1.10%, 0.90%, 1.23%, and 2.10%, respectively), although a slight decrease was observed following treatment with TQ or AuNPs/TQ (0.63% for both), in contrast to the 0.93% observed in EAC tumor-bearing mice receiving PBS (Fig. 7B). Additionally, treating EAC tumor-challenged mice with CP, TQ, AuNPs, AuNPs/TQ, AuNPs/TQ + CP, AgNPs, or AgNPs/TQ resulted in a decrease in % of late apoptosis of EAC cells (4.13%, 3.90%, 1.53%, 5.17%, 4.53%, 2.47%, and 3.67%, respectively). However, EAC tumor-bearing mice treated with AgNPs/TQ + CP exhibited a notable increase in late apoptosis percentage (8.33%) relative to EAC tumor-challenged mice receiving PBS (5.50%) (Fig. 7C). It is noteworthy that EAC tumor-bearing mice injected with AgNPs/TQ + CP demonstrated a noticeable increase in late apoptosis percentage, while those treated with TQ, AuNPs/TQ, AuNPs/TQ + CP, AgNPs, or AgNPs/TQ displayed a prominent elevation in necrosis percentage relative to EAC tumor-bearing mice that received CP.

### Assessment of the anti-inflammatory approach

**Fig. 8:**
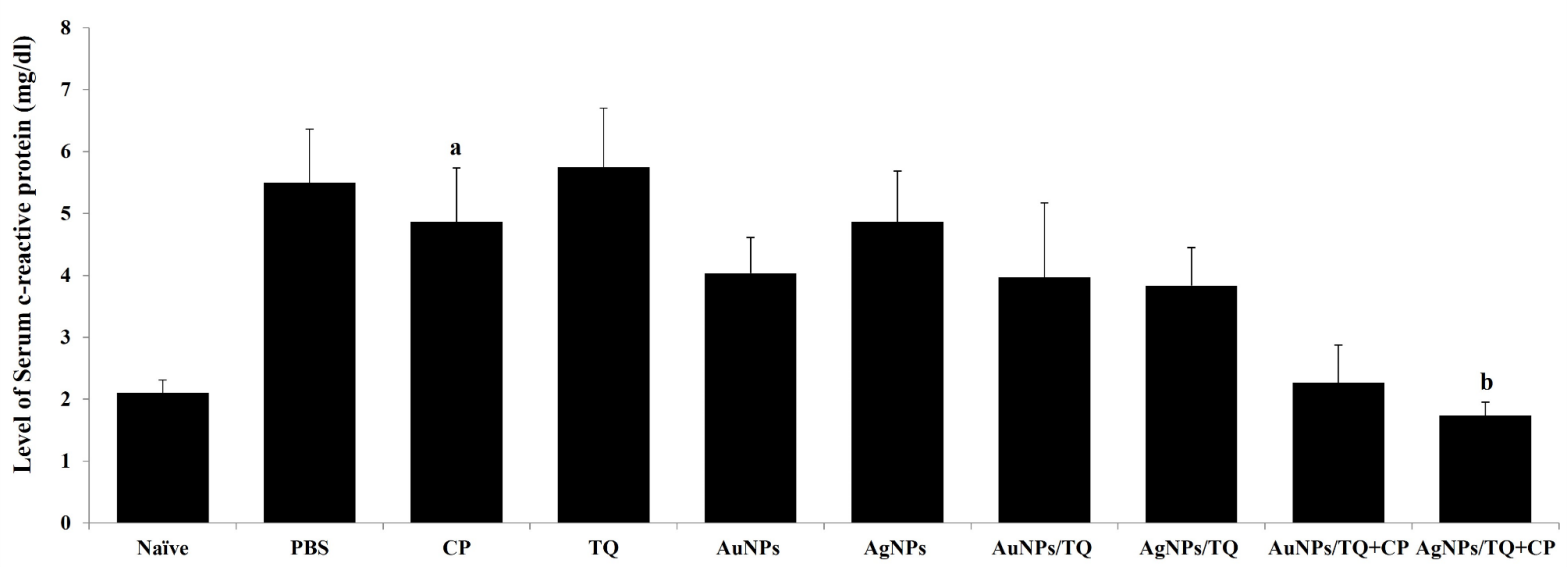
Potentials of AuNPs or AuNPs composites on the level of serum CRP in EAC tumor-bearing mice. EAC-bearing mice were treated with PBS, CP, TQ, AuNPs, AgNPs AuNPs/TQ, ANPs/TQ, AuNPs/TQ + CP, or ANPs/TQ + CP daily for 6 successive days. The euthanasia of the mice occurred on the eleventh day subsequent to the inoculation of tumor cells. EAC cells were collected to assess their viability through the trypan blue method. The samples of sera were collected. Data are stated as mean ± SE (*n* = 5). A statistically significant difference between the groups was established at *P* < 0.05. ^a, b,c^ indicate statistically significant differences when compared to the naïve mice receiving PBS (a), EAC tumor-bearing mice receiving BPS (b)

The findings further indicated that the inoculation of PBS to EAC tumor-bearing mice led to an elevation in serum CRP levels, measuring 5.50 ± 3.28 mg/dl, compared to the naive group, which recorded 2.10 ± 0.49 mg/dl. In contrast, treating EAC tumor-bearing with CP, AuNPs, AuNPs/TQ, AuNPs/TQ + CP, AgNPs, AgNPs/TQ, or AgNPs/TQ + CP significantly decreased serum CRP levels, yielding values of 4.87 ± 1.45 mg/dl, 4.03 ± 1.73 mg/dl, 3.97 ± 1.20 mg/dl, 2.27 ± 0.61 mg/dl, 4.87 ± 1.35 mg/dl, 3.83 ± 0.62 mg/dl, and 1.73 ± 0.22 mg/dl, respectively (Fig. 8). Conversely, treating EAC tumor-bearing mice with TQ caused a prominent elevation in CRP levels, reaching 8.5 ± 1.7 mg/dl, relative to the PBS-treated EAC tumor-bearing mice, which had a CRP level of 5.50 ± 3.28 mg/dl (Fig. 8).

### Analysis of immune cells profile

The analysis of the total leucocyte count following the administration of TQ, AuNPs, AuNPs/TQ, AuNPs/TQ + CP, AgNPs, or AgNPs/TQ to EAC tumor-bearing mice revealed a prominent elevation in the overall white blood cell count (5466.7 ± 1633, 7933.3 ± 966.6, 6166.7 ± 1364, 5666.7 ± 1328, 7100 ± 1159, 5133 ± 785, respectively). In contrast, a reduction in the total leucocyte count was observed in EAC tumor-bearing mice treated with CP or AgNPs/TQ + CP (4500 ± 945, 3666 ± 775, respectively) relative to EAC tumor-bearing mice receiving PBS (4633.3 ± 294.4 × 10^3) and naïve mice (4800 ± 1069) (Table 2). Furthermore, treatment with CP, AuNPs, AuNPs/TQ, AuNPs/TQ + CP, AgNPs, or AgNPs/TQ significantly raised the absolute neutrophil count (107 ± 39.5, 1033 ± 331.6, 250 ± 96.4, 295.3 ± 136.9, 307 ± 105.7, 520.6 ± 66.2, respectively). Conversely, EAC tumor-bearing mice receiving PBS, TQ, or AgNPs/TQ + CP exhibited a drop in the absolute neutrophil count (61.3 ± 14.5, 91 ± 2.5, 92.6 ± 6.6) relative to naïve mice (96.0 ± 21.4) (Table 2).

**Table 2:** Changes in the total number of the leucocyte indices and the absolute number of their differentials in EAC tumor-bearing mice receiving CP, TQ, AuNPs, AgNPs AuNPs/TQ, AgNPs/TQ, AuNPs/TQ + CP, and AgNPs/TQ + CP

Treatments	WBCs count (× 10^3^)	Leucocytes differentials relative number (%)		
		Lymphocytes	Neutrophils	Monocytes
Naïve	4800.0 ± 1069	4668.30 ± 1065	96.0 ± 21.4	25.67 ± 13.74
PBS	4633.3 ± 294	4525.67 ± 240	61.3 ± 14.5	46.33 ± 2.40
CP	4500.0 ± 945	4328.00 ± 947	107.0 ± 39.5	51.33 ± 36.93
TQ	5466.7 ± 1633	5310.70 ± 1626	91.0 ± 2.5	54.67 ± 16.33
AuNPs	7933.3 ± 966	6064.00 ± 253	1033.0 ± 331a, b,c	569.33 ± 228b, c
AgNPs	7100.0 ± 1159	7755.00 ± 303	307.0 ± 105	152.67 ± 56.1
AuNPs/TQ	6166.7 ± 1364	5855.00 ± 1255	250.0 ± 96.4	61.67 ± 13.64
AgNPs/TQ	5133.0 ± 785	4446.60 ± 810	520.6 ± 66	102.67 ± 15.72
AuNPs/TQ + CP	5666.7 ± 1328	5231.30 ± 1146	295.3 ± 136	113.33 ± 26.57
AgNPs/TQ + CP	3666.0 ± 775	3484.00 ± 767	92.6 ± 6.6	54.67 ± 14.50

Data are postulated as mean ± SE (*n* = 5). A statistically significant difference between the groups was established at *P* < 0.05. ^a, b,c^ indicate statistically significant differences, when compared to the naïve mice group (a), EAC tumor-bearing mice receiving PBS (b), EAC tumor-bearing receiving CP (c)

Additionally, EAC tumor-bearing mice receiving TQ, AuNPs, AuNPs/TQ, AuNPs/TQ + CP, AgNPs, or AgNPs/TQ exhibited a remarkable increase in the relative number of lymphocytes compared to naïve mice, with values recorded as follows: 5310.7 ± 1626.8, 6064 ± 253, 5855 ± 1255.3, 5231.3 ± 1146.2, 7755 ± 303, and 4446.6 ± 810, respectively. In contrast, EAC tumor-bearing mice receiving CP or AgNPs/TQ + CP showed a reduction in lymphocyte counts, with values of 4328.00 ± 947 and 3484.00 ± 767, respectively, relative to EAC tumor-bearing mice receiving PBS (4525.67 ± 240.2) and naïve mice (4668.30 ± 1065) (Table 2).

Furthermore, EAC tumor-bearing mice that received injections of CP, TQ, AuNPs, AuNPs/TQ, AuNPs/TQ + CP, AgNPs, AgNPs/TQ, or AgNPs/TQ + CP showed a marked enhancement in the relative count of monocytes, with counts of 51.33 ± 36.93, 54.67 ± 16.33, 569.33 ± 228.4, 61.67 ± 13.64, 113.33 ± 26.57, 152.67 ± 56.1, 102.67 ± 15.72, and 54.67 ± 14.50, respectively. This is in comparison to EAC-bearing mice receiving PBS (46.33 ± 2.40) and naïve mice (25.67 ± 13.74) (Table 2).

### Analysis of kidney and liver functions

The findings indicated that EAC tumor-bearing mice received PBS exhibited a reduction in serum ALT levels (45.00 ± 2.31 U/L) alongside an elevation in serum AST levels (65.00 ± 3.21 U/L) compared to the naive group (87.33 ± 7.22 U/L for ALT and 57.67 ± 10.11 U/L for AST). In contrast, treatment with CP, TQ, AuNPs, AuNPs/TQ, AuNPs/TQ + CP, AgNPs, AgNPs/TQ, or AgNPs/TQ + CP significantly lowered serum ALT levels (29 ± 1.05 U/L, 16.3 ± 0.88 U/L, 29 ± 2.05 U/L, 12.3 ± 0.33 U/L, 22 ± 3.6 U/L, 26.6 ± 4.9 U/L, 22.6 ± 5.3 U/L, 28.6 ± 4.05 U/L respectively) relative to the PBS-treated group (45.00 ± 2.31 U/L) (Table 3). Additionally, the administration of CP, TQ, AuNPs/TQ, AgNPs/TQ, or AgNPs/TQ + CP to EAC tumor-challenged mice resulted in decreased serum AST levels (56.33 ± 0.88 U/L, 41.00 ± 5.86 U/L, 45.00 ± 2.65 U/L, 60.33 ± 9.06 U/L, 56.00 ± 2.65 U/L respectively). However, treatments with AuNPs, AuNPs/TQ + CP, or AgNPs caused a prominent elevation in serum AST levels (84.00 ± 8.33 U/L, 90.00 ± 10.41 U/L, 110.00 ± 10.41 U/L individually) (Table 3).

**Table 3:** Changes in the level of serum AST, and ALT of EAC tumor-bearing mice receiving CP, TQ, AuNPs, AgNPs AuNPs/TQ, AgNPs/TQ, AuNPs/TQ + CP, and AgNPs/TQ + CP

Groups	ALT (U/L)	AST (U/L)
Naïve	87.33 ± 7.22	57.67 ± 10.11
PBS	45.0 ± 2.31^a^	65.00 ± 3.21
CP	29.0 ± 0.58^a, b^	56.33 ± 0.88
TQ	16.33 ± 0.88^a, b^	41.00 ± 5.86
AuNPs	29.0 ± 0.58^a, b^	84.00 ± 8.33
AgNPs	26.67 ± 4.91^a, b^	110.00 ± 10.41^a, b,c^
AuNPs/TQ	12.33 ± 0.33^a, b,c^	45.00 ± 2.65
AgNPs/TQ	22.67 ± 5.36^a, b^	60.33 ± 9.06
AuNPs/TQ + CP	22.0 ± 3.61^a, b^	90.00 ± 10.41^a, c^
AgNPs/TQ + CP	28.67 ± 4.06^a, b^	56.00 ± 2.65

Data are postulated as mean ± SE (*n* = 5). A statistically significant difference between the groups was established at *P* < 0.05. ^a, b,c^ indicate statistically significant differences, when compared to the naïve mice group (a), EAC tumor-bearing mice receiving PBS (b), EAC tumor-bearing receiving CP (c)

EAC tumor-bearing mice receiving PBS treatment exhibited raised serum levels of Urea (39.30 ± 0.88 mg/dl) and Creatinine (0.71 ± 0.03 mg/dl) relative to the naive group, which showed levels of 79.00 ± 3.06 mg/dl and 1.88 ± 0.12 mg/dl, respectively. In contrast, the administration of CP, TQ, AuNPs/TQ, AgNPs, or AgNPs/TQ resulted in significant increases in serum Urea levels, recorded at 55.00 ± 0.58 mg/dl, 51.00 ± 4.16 mg/dl, 57.00 ± 1.16 mg/dl, 42.00 ± 1.73 mg/dl, and 40.00 ± 2.65 mg/dl, respectively. However, treatment with AuNPs or AuNPs/TQ + CP and AgNPs/TQ + CP did not alter or lead to a decrease in serum Urea levels (38.00 ± 2.08 mg/dl, 38.00 ± 3.61 mg/dl, and 26.00 ± 1.53 mg/dl, respectively) relative to the PBS-treated group (39.30 ± 0.88 mg/dl) (Table 4). Furthermore, treatment with CP, TQ, AuNPs, AuNPs/TQ, AuNPs/TQ + CP, AgNPs, AgNPs/TQ, or AgNPs/TQ + CP resulted in a prominent elevation in serum creatinine levels (2.37 ± 0.09 mg/dl, 1.05 ± 0.03 mg/dl, 0.80 ± 0.03 mg/dl, 1.47 ± 0.26 mg/dl, 0.95 ± 0.03 mg/dl, 1.68 ± 0.13 mg/dl, 1.15 ± 0.13 mg/dl, and 2.15 ± 0.10 mg/dl, respectively) relative to the PBS-received group (0.71 ± 0.03) (Table 4).

**Table 4:** Changes in the level of serum creatinine, and Urea of EAC tumor-bearing mice receiving CP, TQ, AuNPs, AgNPs AuNPs/TQ, AgNPs/TQ, AuNPs/TQ + CP, and AgNPs/TQ + CP

Groups	Urea (mg/dl)	Creatinine (mg/dl)
Naïve	79.00 ± 3.06	1.88 ± 0.12
PBS	39.30 ± 0.88^a^	0.71 ± 0.03^a^
CP	55.00 ± 0.58 ^a, b^	2.37 ± 0.09 ^b^
TQ	51.00 ± 4.16 ^a, b^	1.05 ± 0.03^a, c^
AuNPs	38.00 ± 2.08^a, c^	0.80 ± 0.03 ^a, c^
AgNPs	42.00 ± 1.73 ^a, c^	1.68 ± 0.13 ^b, c^
AuNPs/TQ	57.00 ± 1.16 ^a, b^	1.47 ± 0.26 ^b, c^
AgNPs/TQ	40.00 ± 2.65 ^a, c^	1.15 ± 0.13 ^a, c^
AuNPs/TQ + CP	38.00 ± 3.61^a, c^	0.95 ± 0.03 ^a, c^
AgNPs/TQ + CP	26.00 ± 1.53 ^a, b,c^	2.15 ± 0.10 ^b^

Data are postulated as mean ± SE (*n* = 5). A statistically significant difference between the groups was established at *P* < 0.05. ^a, b,c^ indicate statistically significant differences, when compared to the naïve mice group (a), EAC tumor-bearing mice receiving PBS (b), EAC tumor-bearing receiving CP (c)

## Discussion

Resistance to anti-tumor therapies presents considerable obstacles in cancer therapy, contributing significantly to the failure of chemotherapy. This underscores the ongoing necessity for the creating of innovative, effective, and cost-efficient anti-tumor treatments [53, 129, 132]. Given the toxic nature and lack of selectivity associated with many chemotherapeutic agents [31, 117], there is a growing preference for natural therapeutics, which are favored for their reduced toxicity and greater acceptability [98]. Consequently, the potential of TQ to improve the therapeutic effectiveness of anti-cancer therapies while safeguarding normal tissues from chemotherapy-related damage has gained attention [19]. The pathway through which nanocarriers are internalized can significantly influence their subsequent behavior post-internalization. Therefore, encapsulating therapeutic agents within NPs that possess a specific affinity for these pathways may improve the efficacy of membrane targeting [69, 134], thereby facilitating increased uptake by cancer cells [14, 39, 57, 102]. Currently, AuNPs and AgNPs are the focus of extensive research due to their comparatively low toxicity to normal cells when contrasted with other metal NPs [56].

The findings from these in vivo experiments were consistent with our in vitro results, indicating that the conjugates of AuNPs, AgNPs, AuNPs/TQ, AuNPs/TQ + CP, AgNPs/TQ, and AgNPs/TQ + CP have the potential to specifically target CP to tumor tissues. Furthermore, they are capable of releasing free drugs in the environment characteristic of tumors, thereby enhancing their efficacy in antitumor drug delivery. In vivo as in vitro results, AuNPs/TQ, AgNPs/TQ, AuNPs/TQ + CP, and AgNPs/TQ + CP showed stronger anti-tumor efficacy than free CP and were tumor-targeting.

Our in vivo findings indicate that AuNPs, AuNPs/TQ, AuNPs/TQ + CP, AgNPs, AgNPs/TQ, or AgNPs/TQ + CP can specifically target tumor tissues, suppress tumor growth, extend the lifespan of tumor-bearing mice, and minimize cytotoxic effects on normal tissues, relative to the administration of free CP alone in vivo.

The present investigations aimed to explore the anti-tumor effects of AuNPs/TQ conjugate or AgNPs/TQ conjugate, both with and without the classical anticancer agent CP, assessing the potential anticancer therapeutic effects of these composites when administered orally to EAC tumor-bearing mice. The current outcomes indicate that the effective penetration characteristics of the mixtures of AuNPs and AgNPs significantly inhibited the proliferation rate of EAC cells in EAC tumor-bearing mice, in comparison to those treated with PBS, likely due to enhanced uptake.

The integration of AuNPs or AgNPs with TQ significantly enhanced their cytotoxic and anti-proliferative effects against EAC cells in EAC tumor-bearing mice, resulting in increased percentages of necrosis and apoptosis among the tumor cells. These findings align with the research conducted by [89], who suggested that synthesized nanoparticles could effectively inhibit the mobilization of breast cancer, leading to a marked reduction in tumor growth rate and volume, thereby extending the lifespan of the treated subjects. Furthermore, NPs promote cancer cell apoptosis via mechanisms that include the mediation of reactive oxygen species (ROS), immunological responses, modulation of protein expression, transcriptional inhibition, and targeted cytotoxicity [91]. Furthermore, the primary advantage of TQ as an antineoplastic agent lies in its capacity to disrupt cancer cell proliferation while presenting minimal or no risk to healthy cells [121]. The regulated release of TQ from AgNPs, which accumulate in tumor tissue, minimizes exposure to normal tissues and mitigates the rapid metabolism of the administered drug [77]. TQ encapsulated in AgNPs has shown promising outcomes in delaying the carcinogenesis process and enhancing the overall health of the animals, as indicated by a notable reduction in p53 nuclear expression compared to free TQ [112].

AuNPs and AgNPs exhibit significant cytotoxic effects on cancerous cells and demonstrate a strong affinity for biological macromolecules. This characteristic enables them to effectively traverse cellular barriers, thereby reducing cancer cell proliferation [51, 63, 133, 141]. The resulting apoptosis of cancer cells is chiefly attributed to ROS, which inflicts damage on cellular components through intracellular oxidative stress, finally causing cell death [105, 126]. Furthermore, alterations in mitochondrial membrane potential result in the liberation of cytochrome c, triggering mitochondrial-related apoptosis and necrosis, which further inhibits cell proliferation and carcinogenesis [34]. AgNPs, characterized by their small size and large surface area, possess the capability to penetrate tissues and cells, generating ROS that inhibits antioxidant systems within the blood and tissues of mice [40, 81, 86]. Furthermore, AgNPs exhibit anticancer properties by inducing membrane permeability and nuclear condensation in an apoptotic manner, facilitated by the activation of caspases and the production of ROS [109]. Additionally, Ag + ions may activate the P53 pathway and other cell cycle regulatory genes, preventing cells from progressing to the M phase and promoting cell death through apoptosis [15, 47].

AgNPs have demonstrated significant anti-angiogenic and anti-proliferative effects on cancer cells. This is contributed to their capacity to inflict DNA damage, induce chromosomal breaks, create genomic instability, and disrupt calcium (Ca2+) homeostasis, which collectively triggers apoptosis and leads to cytoskeletal instability. These processes hinder the cell cycle and division, thereby enhancing the anti-proliferative effects on cancer cells [16, 55, 138]. Furthermore, the anticancer efficacy of AgNPs is augmented by their radical scavenging activity, where an increase in free radical scavenging correlates with heightened cytotoxicity [106, 111]. This effect is complemented by a reduction in mitochondrial function, the release of lactate dehydrogenase, chromosomal abnormalities, DNA damage, the enhancement of apoptotic genes, cell cycle deregulation, and the synthesis of micronuclei [54, 81].

AuNPs possess the capability to act as antiangiogenic agents by inhibiting signaling pathways and normal cellular functions, as well as serving as anticancer agents due to their photodynamic and photothermal properties. They also impede the phosphorylation of downstream molecules [108]. Furthermore, AuNPs exhibit a selective affinity for cancer cells, promoting apoptosis by influencing tumor-suppressor genes and oncogenes, which augments the expression of caspase-9, thereby facilitating the apoptotic process [128]. This effect may also result from the disruption of the cell cycle and the inhibition of cytokinesis when AuNPs target the nucleus [67]. Additionally, AuNPs have confirmed efficacy in in vivo phototherapy, attributed to their substantial absorption in the infrared region of the electromagnetic spectrum and their capacity to generate localized heat that can eliminate targeted areas [61]. Moreover, AuNPs and AgNPs are emerging as promising anticancer agents due to their effectiveness against drug-resistant tumor cells, making it challenging for resistance to develop. Their nanoscale size facilitates targeted delivery by enabling them to traverse the fenestrations in blood capillaries and even the blood-brain barrier [32]. Furthermore, TQ has exhibited potent antineoplastic activity on cancerous cells with minimal or no adverse effects on healthy cells [121]. The release of TQ from AuNPs that aggregate within cancerous tissues will minimize exposure to normal tissues, thereby disrupting the rapid metabolism of the administered drug [77, 112].

The data collected here revealed that treating EAC tumor-bearing mice with TQ, AuNPs, AuNPs/TQ, AgNPs, or AgNPs/TQ showed no substantial change or only a minor increase in splenocyte counts. In contrast, the administration of CP to EAC tumor-bearing mice caused a notable reduction in splenocyte numbers compared to EAC-tumor mice receiving PBS. Additionally, the administration of AuNPs, AgNPs, AuNPs/TQ, and AgNPs/TQ to EAC tumor-bearing mice resulted in a reduction of serum CRP levels. The addition of CP to the AuNPs/TQ or AgNPs/TQ conjugates significantly lowered CRP levels to within the normal range, as observed in naive mice receiving PBS. This finding aligns with previous studies of [73, 82, 84], which suggest that NPs likely interact with the innate immune system upon entry into the body, potentially eliciting an immunomodulatory response influenced by their physicochemical properties. Both AgNPs and AuNPs can stimulate lymphocyte activity in vitro, which may pose a risk to immune function in situ [37]. Additionally, AgNPs and AuNPs exhibit anti-inflammatory properties by inhibiting the discharge of pro-inflammatory mediators in LPS-stimulated RAW264.7 cells [119]. Moreover, AgNPs and AuNPs have demonstrated immunomodulatory effects and the capability to induce apoptosis [46].

Furthermore, AgNPs can decrease the accumulation of inflammatory cells and the production of inflammatory cytokines through the upregulation of tumor growth factor- β1 (TGF-β1) and interleukin-10 (IL-10) [9]. In vivo, AgNPs exert anti-inflammatory effects by inhibiting nuclear factor-κB (NF-κB) and pro-inflammatory molecules while promoting the expression of anti-inflammatory molecules. They are subsequently inactivated by intracellular glutathione (GSH), which minimizes cytotoxicity in healthy cells. This process caused a decrease in CRP produced by the liver, and the suppression of this protein represents an effective approach to managing inflammation without disrupting the immune system or cellular homeostasis [11, 13, 26, 41]. Additionally, AgNPs can alleviate inflammation by lowering edema and reducing IL-6 levels and high-sensitive CRP (hs-CRP) [68, 101].

The present study indicated that there were not many significant variations, whether increasing or decreasing, in the total leukocyte counts and the absolute numbers of neutrophils, lymphocytes, monocytes, and eosinophils following treatment with TQ, AuNPs, AuNPs/TQ, AuNPs/TQ + CP, AgNPs, or AgNPs/TQ, AgNPs/TQ + CP. This suggests that these conjugates may induce a homeostatic condition characterized by a decrease in progenitor cell frequency without adversely affecting the production of total leukocytes, neutrophils, lymphocytes, monocytes, and eosinophils within the myeloid compartment of the bone marrow. Consistent with our findings, Bain [20] demonstrated that various herbal phytoconstituents influence the immune system by modulating the activity of several immune cells, including total leukocytes, neutrophils, monocytes, eosinophils, macrophages, basophils, and natural killer cells, while also regulating phagocytic cells [58, 72, 103, 33]. The immunomodulatory properties of natural products may serve as promising therapeutic targets for the progress of new immunomodulatory drugs that enhance chemotherapy by restoring immune surveillance and addressing the complex immunosuppressive tumor microenvironment, which poses significant challenges in the creation of effective cancer therapies [12]. Furthermore, these products activate innate immune components such as neutrophils, lymphocytes, monocytes, and eosinophils [114, 122].

NPs are likely to interact with immune cells within the body, leading to significant interactions between NPs and the immune system, which may exhibit immunomodulatory effects by either activating or suppressing immune responses [115, 120, 140, 73]. AuNPs exhibited no cytotoxicity towards mouse macrophages and selectively suppressed Cytokines that are inhibited with the pathogenic immune response mediated by T helper 17 cells (Th17) cells, potentially through the controlling of ROS, indicating that AuNPs may possess immune-modulatory properties that could help mitigate inflammatory or pathogenic immune responses [70]. Neither AgNPs nor AuNPs coated with amphiphilic polymers exhibited significant harmful effects on hematological parameters [62]. The elevation in leucocytes following AgNP exposure is likely a normal immune response in rats, resulting from the immune system’s activation to facilitate the phagocytosis of AgNPs or could be linked to stress responses triggered by AgNPs administration [60]. Consequently, AgNPs AgNPs can have a considerable impact on the ratios of lymphocytes to granulocytes [18]. AuNPs can inhibit the phagocytic and chemotactic activities of macrophages and polymorphonuclear leukocytes (PMN), as well as interfere with the initial component of the complement system, disrupt prostaglandin synthesis, and inhibit lysosomal enzymes that may contribute to inflammation [35, 95, 125].

The outcomes of the ongoing research indicated notable variations in liver function, as indicated by levels of ALT and AST, as well as kidney function, reflected in levels of creatinine and urea, in EAC tumor-challenged mice, received TQ, AuNPs, AuNPs/TQ, AuNPs/TQ + CP, AgNPs, or AgNPs/TQ, AgNPs/TQ + CP, when compared to control EAC tumor-challenged mice receiving only PBS. These results align with those reported by [62, 78], who demonstrated that PEGylated Au NPs did not exhibit significant effects on endothelial cells following their entry into the bloodstream and that liver-damaging effects from amphiphilic polymer-coated AgNPs and AuNPs were not observed. TQ was observed to reduce the elevation of serum markers associated with kidney and liver functions induced by AgNPs, including creatinine, urea, alanine transaminase, and aspartate transaminase. Furthermore, the co-administration of TQ with AgNPs diminished oxidative damage to the liver and kidneys by lowering levels of MDA and NO, while significantly enhancing antioxidant enzyme activity. Additionally, the co-administration of TQ suggested a potential mechanism for its antioxidants, anti-inflammatory, and anti-apoptotic properties, as it also reduced levels of pro-inflammatory mediators in both liver and kidney, indicating that TQ offers protection against AgNPs cytotoxicity [110]. Moreover, the oral administration of thymoquinone-solid lipid nanoparticles resulted in decreased serum biomarker enzyme levels (AST & ALT) compared control group (paracetamol-induced liver cirrhosis). The systemic administration of AuNPs showed no changes to urea and creatinine levels, which are prompted by the size of the AuNPs [3].

The impact of orally administered AgNPs on the activities of AST and ALT did not exhibit a consistent trend, suggesting a potential partial inactivation of enzyme function or a reduction in enzyme synthesis. The liver is frequently identified as a primary target organ and a major site for the accumulation of nanoparticles. The observed changes in enzyme levels may indicate adaptive responses by the organisms to mitigate the stress induced by AgNPs exposure [135]. The attraction of AgNPs to thiol groups may influence their ability to regulate enzyme activity, as the occurrence of these groups in enzymes may facilitate the AgNPs binding, causing the formation of complexes that subsequently influence enzyme activity. Notably, AgNPs caused an incredible drop in the level of ALT and a notable elevation in the level of AST levels following 21 days of treatment [4–6]. The noted reduction in AST activity could be ascribed to the inactivation of proteins or enzymes resulting from the affinity of AgNPs for thiol groups (-SH) groups. Such inactivation could have detrimental effects on cellular integrity, as it may disrupt essential metabolic processes [1, 123]. Furthermore, exposure to Ag/Au NPs caused significant changes in serum creatinine and urea levels, in addition to alterations in ALT and AST activities [124].

NPs represent a highly effective option for addressing hepato-renal deficiencies due to their pro-oxidant, antioxidant, and anti-inflammatory characteristics. Furthermore, systemic indicators of diminished liver inflammation were noted, as demonstrated by the reduction in AST and ALT enzyme levels [59]. Given that AuNPs-TQ does not adversely affect liver or kidney enzymes, it is viewed as a promising chemotherapeutic agent for cancer treatment. AuNPs-TQ is recognized as a viable chemotherapeutic option for carcinoma, exhibiting no toxic effects on liver and kidney enzymes [42]. The noted reduction in AST activity could be ascribed to the inactivation of proteins or enzymes resulting from the affinity of AgNPs for thiol groups., including the heart, lungs, and kidneys. Additionally, the circulation and excretion patterns of NPs in the bloodstream vary between AgNPs and AuNPs [119]. AuNPs have shown potential in mitigating oxidative stress to alleviate oxidative stress and mitigate histological damage within the renal tissues, with their capacity to scavenge free radicals believed to contribute to their therapeutic benefits [7, 23]. Moreover, AuNPs promote the restoration of impaired mitochondria, improve the elimination of ROS from these organelles, and modulate antioxidant concentrations within the brain [8].

AuNPs and AgNPs present numerous benefits as drug delivery systems, but their safety remains the primary limitation to broader applications. Most studies suggest that AuNPs and AgNPs are non-toxic; however, some research has revealed instances of toxicity. This poses a potential issue when employing metal NPs in cancer therapy, as they can harm healthy cells alongside cancerous ones [27]. Factors contributing to toxicity may include the NPs’ size, shape, the materials they are conjugated with, their dosage, and their biodegradability. Given these limitations, there is a pressing need for more comprehensive and standardized criteria to assess the toxicity of AuNPs and AgNPs. An outstanding drawback of AuNPs and silver AgNPs is the possibility of metal NPs accumulating in the body over an extended period. Research indicates that these NPs can gather in various organs, including the spleen and liver, which may lead to long-term health implications [50]. Therefore, it is essential to monitor and mitigate this accumulation to guarantee the safety of utilizing NPs. Additionally, there is a pressing need to refine the surface chemistry, shape, and size of these NPs to enhance their efficacy in tumor diagnosis and therapy [45]. Variations in these characteristics can influence their interactions with cancer cells, resulting in differing levels of effectiveness across various cancer types. Consequently, optimizing these parameters for the utilization of metal NPs in cancer care remains a vital focus of ongoing research [44]. Other challenges hindering the clinical application of NPs in cancer treatment include issues related to insufficient biodegradation and removal, protracted drug development timelines, difficulties in achieving effective drug loading within nanoparticles, obstacles in drug incorporation and release, challenges in cellular uptake, and the inability to translate in vitro findings to in vivo scenarios [44]. Therefore, more precise techniques for the targeted delivery of NPs to sites of cancer growth are required.

## Conclusion

To conclude, AuNPs, AgNPs, AuNPs/TQ, and AgNPs/TQ may hold great promise as a potential NP-based delivery for cancer treatment. Furthermore, they provide various benefits compared to conventional cancer treatments, including enhanced selectivity and reduced side effects. Additionally, AuNPs, AuNPs/TQ, AuNPs/TQ + CP, AgNPs, AgNPs/TQ, or AgNPs/TQ + CP can specifically target tumor tissues, suppress tumor growth, extend the lifespan of tumor-bearing mice, and minimize cytotoxic effects on normal tissues, relative to the administration of free CP alone in vivoAdditional research is crucial to elucidate the mechanisms that govern these nanoparticle-based therapies in clinical settings and to optimize their application in cancer treatment.

## Data Availability

The data that support the findings of this study are available from the corresponding author upon reasonable request.

## Abbreviations

TQ: Thymoquinone
CP: Cisplatin
AuNPs: Gold nanoparticles
AgNPs: Silver nanoparticles
EAC: Ehrlich ascites carcinoma
